# Exosomes from IL-33-Stimulated Macrophages Regulate Epithelial Barrier Function to Ameliorate TNBS-Induced Colitis in Mice

**DOI:** 10.3390/cells15131217

**Published:** 2026-07-03

**Authors:** Shuang Liu, Ye Cao, Luhui Chen, Qianying Nie, Wanxia Liu, Yu Zhao, Baohong Yuan, Tao Liu, Ying Liu, Hui Yin

**Affiliations:** 1 School of Basic Medical Sciences, Guangdong Pharmaceutical University, Guangzhou 510006, China; 2112440142@stu.gdpu.edu.cn (S.L.); 2112440140@stu.gdpu.edu.cn (Y.C.); 2112458011@stu.gdpu.edu.cn (L.C.); nqy5788@163.com (Q.N.); wanx_liu@163.com (W.L.); 1112526005@stu.gdpu.edu.cn (Y.Z.); yuanbaohong@gdpu.edu.cn (B.Y.); tliu@foxmail.com (T.L.); 2 School of Pharmacy, Guangdong Pharmaceutical University, Guangzhou 510006, China

**Keywords:** exosomes, Interleukin-33, inflammatory bowel disease, Wnt/β-catenin, macrophage

## Abstract

Inflammatory bowel disease (IBD) represents a growing global health threat that markedly increases colorectal cancer risk, yet conventional immunosuppressive agents achieve mucosal healing in only a limited subset of patients. M2-polarized macrophages have been recognized as crucial regulators of mucosal repair through their ability to maintain intestinal microenvironment homeostasis. Here, we investigated the potential effects and mechanisms of macrophage-derived exosomes (Exos) on epithelial barrier function in a murine model of IBD. Murine colitis was induced by intrarectal administration of 2,4,6-trinitrobenzene sulfonic acid (TNBS), followed by treatment with Exos isolated from IL-33-treated macrophages (IL-33-Exos) or untreated macrophages (PBS-Exos). Our findings showed that IL-33-Exos markedly ameliorated inflammatory intestinal mucosal injury and improved intestinal barrier dysfunction. Concurrently, IL-33-Exos mitigated intestinal epithelial cell damage, thereby preserving intestinal mucosal integrity. Mechanistic studies revealed that the beneficial effects of IL-33-Exos were implicated in upregulation of Wnt/β-catenin signaling in intestinal epithelial cells. Translationally, these findings suggest that IL-33-Exos may promote epithelial repair in experimental colitis, offering a novel therapeutic avenue for clinical management of inflammatory bowel disease.

## 1. Introduction

The prevalence of inflammatory bowel disease (IBD) continues to escalate globally, with a concomitant increase in colorectal cancer risk, rendering IBD a significant and growing healthcare burden [1]. The etiology and pathogenesis of IBD are multifactorial, involving complex interactions among aberrant immune function, environmental factors, compromised colonic epithelial integrity, and dysbiosis of the intestinal microbiota [2]. The intestinal microbiota of patients with IBD is altered, with a significant loss of a variety of short-chain fatty acids (SCFAs) produced by commensal bacteria [3]. Current clinical therapeutics for IBD, including 5-aminosalicylic acid, anti-TNF-α agents, and novel small-molecule Janus kinase inhibitors, primarily target immune homeostasis restoration. However, these treatments demonstrate limited efficacy, achieving mucosal healing in only 30–50% of patients [4,5,6]. Consequently, the identification of effective therapeutic strategies to manage the disease and enhance quality of life for IBD patients has emerged as a pressing medical imperative.

IL-33 serves as a critical anti-inflammatory cytokine that orchestrates wound healing, inflammation resolution, and tissue repair by promoting macrophage polarization toward the M2 phenotype and enhancing regulatory T cell (Treg) activation [7,8,9]. Moreover, immune cells such as T cells, innate lymphoid cells (ILCs), dendritic cells, and macrophages, along with their secreted cytokines, exert essential functions in driving intestinal stem cell (ISC) and intestinal epithelial cell (IEC) regeneration [10,11]. Notably, TLR-mediated macrophage activation and subsequent engraftment mitigate intestinal injury, with macrophages transmitting regenerative signals to ISCs that are indispensable for stem cell self-renewal and proliferation [12]. The crosstalk between macrophages and epithelial cells has been shown to accelerate intestinal mucosal repair [13]. Additionally, butyrate-induced M2 macrophages markedly upregulate mucin-2 (MUC2) and SPDEF (a goblet cell marker), activate WNT/ERK signaling, and foster mucosal restoration following ulcerative colitis (UC)-induced damage [14]. However, the precise impact of IL-33-primed macrophages on IEC regeneration and barrier integrity remains to be fully elucidated.

Emerging evidence indicates that macrophages secrete exosomes through paracrine mechanisms, thereby participating in diverse physiological and pathological processes, including tumorigenesis, inflammation, and tissue repair [15,16,17]. Exosomes, a subset of extracellular vesicles ranging from 30 to 150 nm in diameter, function as natural nanocarriers that transport bioactive cargoes such as proteins, lipids, and nucleic acids to recipient cells. This intercellular communication modality holds considerable promise for both disease diagnosis and therapeutic applications. Exosome-based therapeutics offer distinct advantages. They serve as efficient drug delivery vehicles, their presence in virtually all body fluids enables noninvasive or minimally invasive biomarker development for cancer detection, and their acellular nature circumvents complications associated with cell-based therapies, notably immune rejection [18,19]. Functionally, M2 macrophage-derived exosomes (M2-Exos) suppress proinflammatory cytokine production, expand Treg cell populations, and attenuate colitis severity in dextran sulfate sodium (DSS)-treated mice [20]. Collectively, these properties position macrophage-derived exosomes as a compelling cell-free alternative to conventional macrophage therapy, with substantial translational potential for inflammatory bowel disease (IBD)-associated mucosal injury repair.

In the present study, we evaluated the therapeutic potential of exosomes derived from IL-33-induced macrophages (IL-33-Exos) in a mouse model of TNBS-induced colitis.

## 2. Materials and Methods

### 2.1. Macrophage Culture

RAW264.7 macrophages (Type Culture Collection of the Chinese Academy of Sciences, Shanghai, China) were maintained in DMEM supplemented with 10% fetal bovine serum and 1% penicillin–streptomycin (Invitrogen, Carlsbad, CA, USA). After washing with PBS, the cells were incubated in serum-free medium and stimulated with 100 ng/mL IL-33 for 24 h. The expression of Wnt ligand-related genes was then quantified by qPCR.

### 2.2. Macrophage Exosome Extraction and Identification

Exosomes were isolated from the culture supernatants of both untreated RAW264.7 cells (PBS-Exos) and IL-33-stimulated RAW264.7 cells (IL-33-Exos) via polyethylene glycol (PEG) precipitation followed by ultracentrifugation, as previously described [21]. The protein concentration of purified exosomes was measured using a BCA assay kit (Beyotime, Shanghai, China). Particle size distribution was characterized by nanoparticle tracking analysis (NTA), while morphological features were examined by transmission electron microscopy. Exosomal identity was further confirmed by Western blot detection of canonical markers (CD63, TSG101, HSP70) and the cargo protein Wnt2b. For cellular uptake studies, IL-33-Exos were labeled with Dil and their internalization by Caco-2 cells was visualized under an Olympus BX51 (Olympus America, Inc., Melville, NY, USA) fluorescence microscope.

### 2.3. Establishment of TNBS Colitis Model and Treatment

Male BALB/c mice (5–6 weeks old) were obtained from the Guangdong Medical Laboratory Animal Center. Colitis was induced using a TNBS-based protocol as previously described [22]. In brief, mice were randomly allocated, lightly anesthetized, and a 3.5-F catheter was inserted rectally to a depth of 4 cm proximal to the anus. A single intraluminal instillation of 150 µL TNBS solution (2.5 mg in 50% ethanol; Sigma-Aldrich, St. Louis, MO, USA) was delivered slowly. Control animals received an equivalent volume of 50% ethanol alone. Twenty-four hours post-TNBS administration, mice were intraperitoneally injected with either 200 μg IL-33-Exos or PBS-Exos. In some experiments, mice received combined intraperitoneal treatment with IL-33-Exos and the Wnt/β-catenin pathway inhibitor XAV939 (2.5 mg/kg; MCE). Animals were monitored daily for diarrhea onset, body weight changes, and survival. All animal procedures were approved by the Guangdong Pharmaceutical University Animal Care and Use Committee (approval no. gdpulac2020182).

### 2.4. DAI Score

The disease activity index (DAI) was computed as the cumulative score of three clinical parameters, including body weight change, stool consistency, and fecal occult blood. Scoring was performed independently by two investigators blinded to group allocation. The scoring criteria were as follows: (i) weight change (0 = no change; 1 = 1–5% loss; 2 = 6–10% loss; 3 = 11–20% loss; 4 = >20% loss); (ii) stool consistency (0 = normal; 1 = soft but formed; 2 = soft; 3 = very soft; 4 = watery diarrhea); and (iii) fecal occult blood (0 = negative; 1 = weakly positive; 2 = positive; 3 = visible blood traces; 4 = gross bleeding). The total DAI score, ranging from 0 to 12, represented the sum of these three component scores.

### 2.5. Histology

Mice were anesthetized and subjected to transcardial perfusion with 4% paraformaldehyde in PBS. Colonic tissues were subsequently excised, fixed overnight in the identical fixative, embedded in paraffin, and sectioned at 4 µm thickness. Following standard dewaxing and rehydration procedures, tissue sections were stained with either hematoxylin and eosin (H&E) or Alcian blue–Periodic acid–Schiff (AB-PAS). Histological images were captured under a microscope, and the severity of tissue damage was assessed by blinded histopathological evaluation as described previously [23].

### 2.6. Tissue Immunofluorescence

Immunofluorescence staining was conducted on 4 µm paraffin-embedded sections of mouse colonic tissue. Following rehydration, tissue sections were blocked with 5% bovine serum albumin (BSA) for 30 min at room temperature. Primary antibody incubation was performed overnight at 4 °C using the following antibodies: anti-Ki67 (Abcam, Cambridge, UK), anti-E-cadherin (Sino Biological, Beijing, China), anti-Occludin (Proteintech, Rosemont, IL, USA), or anti-β-catenin (Boster, Wuhan, China). Subsequently, sections were incubated with PE- or FITC-conjugated secondary antibodies (eBioscience, San Diego, CA, USA). After nuclear counterstaining with DAPI, slides were observed on an Olympus BX51 fluorescent microscope.

### 2.7. Caco-2 Cell Culture and Stimulation with Macrophage Exosomes

Caco-2 cells (Type Culture Collection of the Chinese Academy of Sciences, Shanghai, China) were plated at a density of 3 × 10^5^ cells per well in 24-well plates. Cells were pre-incubated with either 35 μg/mL PBS-Exos or IL-33-Exos for 2 h, followed by stimulation with 50 ng/mL TNF-α for an additional 24 h. Cell viability was assessed using the cell counting kit-8 (CCK-8) assay, and proliferative activity was evaluated by BrdU incorporation immunofluorescence. In some experiments, Caco-2 cells were pretreated with 0.5 μg/mL DKK1 (Sino Biological) or 0.32 μg/mL XAV939 for 30 min prior to IL-33-Exos exposure.

### 2.8. Cell Immunofluorescence

Caco-2 cells were fixed and subjected to immunofluorescence staining using anti-E-cadherin (Sino Biological), anti-Occludin (Proteintech), and anti-β-catenin (Boster) primary antibodies, followed by incubation with PE- or FITC-conjugated secondary antibodies (eBioscience). Nuclear visualization was achieved by DAPI counterstaining. For BrdU incorporation assays, BrdU was pulsed 24 h prior to cell harvest, and incorporated BrdU was detected using an anti-BrdU antibody (Abcam).

### 2.9. Cell Viability Assay

Cell viability was assessed using the cell counting kit-8 (CCK-8) assay (Dojindo, Kumamoto, Japan). Caco-2 cells were plated at 1 × 10^3^ cells per well in 96-well plates. Following 2 h pretreatment with 35 μg/mL PBS-Exos or IL-33-Exos, cells were stimulated with 50 ng/mL TNF-α for 24 h. CCK-8 reagent was then added, and cells were incubated for 2 h at 37 °C. Absorbance was measured at 450 nm using a microplate reader (Model 680, Bio-Rad Laboratories, Hercules, CA, USA).

### 2.10. FITC-Dextran Cell Permeability Assay

Caco-2 cells were seeded at 1 × 10^5^ cells per well onto transwell inserts with 0.4 μm pores (Costar, NY, USA) positioned in 24-well plates. Monolayer integrity and permeability were evaluated by measuring the flux of FITC-dextran (4 kDa, Sigma-Aldrich). FITC-dextran (20 μg/mL) was introduced into the apical chamber, and after 2 h of incubation, basolateral aliquots were collected. Fluorescence intensity was quantified at an excitation wavelength of 490 nm and emission of 520 nm.

### 2.11. Western Blotting

Total protein was extracted from colon tissues and cultured cells by homogenization in RIPA buffer supplemented with a protease inhibitor cocktail (Biosharp, Beijing, China). For subcellular fractionation, cytoplasmic and nuclear proteins were isolated using a commercial extraction kit (Beyotime) following the manufacturer’s protocol. Protein samples (30 μg per lane) were separated by SDS-PAGE and electrotransferred onto PVDF membranes (Millipore, Billerica, MA, USA). Membranes were incubated with the following primary antibodies: anti-β-catenin (Boster), anti-Lgr5 (Sino Biological), anti-Wnt2b (Invitrogen), anti-Occludin (Proteintech), anti-Claudin-1 (Proteintech), anti-E-cadherin (Sino Biological), anti-CD63 (Abcam), anti-HSP70 (Abcam), anti-TSG101 (Abcam), and anti-Calnexin (Abcam). Immunoreactive bands were detected by HRP-conjugated secondary antibodies (Abcam). β-actin (Sino Biological) or Histone H3 (Abcam) served as loading controls. Band intensities were quantified using ImageJ software (version 1.53) and normalized to the corresponding loading control.

### 2.12. RNA Extraction and Quantitative Real-Time PCR

Total RNA was isolated using TRIzol reagent (Invitrogen) and transcribed into cDNA with M-MLV reverse transcriptase (Invitrogen). Quantitative PCR was performed using the SYBR Green qPCR kit (Invitrogen) on an ABI PRISM^®^ 7000 Sequence Detection System (Applied Biosystems, Foster City, CA, USA). The primer sequences employed were as follows: Lgr5 (forward: 5′-CCT ACT CGA AGA CTT ACC CAG T-3′, reverse: 5′-GCA TTG GGG TGA ATG ATA GCA-3′); Wnt10a (forward: 5′-GAC CTG AGT AGG AGC TGT GT-3′, reverse: 5′-GGA TGT CGT TGG GTG CTG AC-3′); Wnt9a (forward: 5′-TGC TTT CCT CTA CGC CAT CT-3′, reverse: 5′-CCC AGG AAC TCC TTG ACA AA-3′); Wnt7b (forward: 5′-CGT GTT TCT CTG CTT TGG CG-3′, reverse: 5′-AGT TCT TGC CCG AAG ACG G-3′); Wnt6 (forward: 5′-GCA AGA CTG GGG GTT CGA G-3′, reverse: 5′-CCT GAC AAC CAC ACT GTA GGA G-3′); Wnt5a (forward: 5′-CAA ATA GGC AGC CGA GAG AC-3′, reverse: 5′-CTC TAG CGT CCA CGA ACT CC-3′); Wnt2b (forward: 5′-AGC ACC AGT TCC GTC ACC AC-3′, reverse: 5′-AGC CAC CCC AGT CAA AGT CC-3′); and GAPDH (forward: 5′-TTC ACC ACC ATG GAG AAG GC-3′, reverse: 5′-GGC ATG GAC TGT GGT CAT GA-3′). Expression values were normalized to the housekeeping gene GAPDH using the comparative threshold cycle (CT) method.

### 2.13. Statistical Analysis

Data are expressed as mean ± SEM. Statistical comparisons were performed using Student’s *t*-test, one-way ANOVA, or two-way ANOVA as appropriate. A *p*-value < 0.05 was considered statistically significant.

## 3. Results

### 3.1. Characterization of Exosomes Derived from IL-33-Preconditioned Macrophages

**Figure 1:**
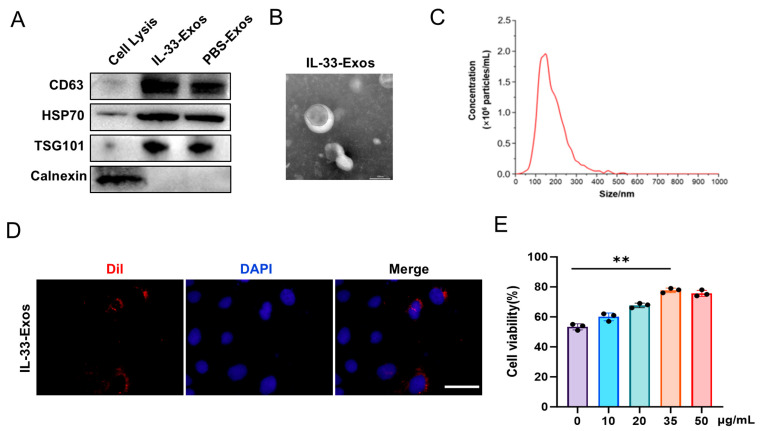
Isolation and characterization of exosomes derived from IL-33-preconditioned macrophages. (**A**) Western blot analysis of CD63, HSP70, TSG101 and Calnexin proteins in exosomes. (**B**) Transmission electron micrograph of macrophage exosomes. Scale bar, 100 nm. (**C**) NTA technology measurement of macrophage exosome particle size distribution. (**D**) Observation of Caco-2 cell uptake of exosome using fluorescence microscopy. Scale bar, 50 μm. (**E**) Assessment of Caco-2 cell viability following IL-33-Exos treatment. Data are mean ± SEM (*n* = 3/group). ** *p* < 0.01.

Macrophage-derived exosomes were isolated from IL-33-stimulated RAW264.7 cells using PEG precipitation combined with ultracentrifugation. The identity and purity of the isolated vesicles were confirmed by multiple approaches. Western blot analysis detected exosome-enriched markers (CD63, HSP70, and TSG101) in both IL-33-Exos and PBS-Exos, with minimal contamination from the endoplasmic reticulum marker Calnexin (Figure 1A). Transmission electron microscopy revealed characteristic cup-shaped morphology with a diameter consistent with exosomal dimensions (Figure 1B). Nanoparticle tracking analysis further validated these findings, showing a predominant size distribution with a peak at approximately 145 nm (Figure 1C). To determine whether these exosomes could be internalized by intestinal epithelial cells, Dil-labeled IL-33-Exos were incubated with Caco-2 cells. Immunofluorescence microscopy demonstrated efficient cellular uptake and perinuclear accumulation (Figure 1D), indicating functional delivery of exosomal cargo to recipient cells. Finally, to assess the biological activity of IL-33-Exos, CCK-8 assay was performed in TNF-α-stimulated Caco-2 cells. IL-33-Exos improved cell viability in a dose-dependent manner, with maximal efficacy observed at 35 μg/mL (Figure 1E). This concentration was subsequently used for the following in vitro experiments.

### 3.2. IL-33-Exos Ameliorate TNBS-Induced Colitis in Mice

**Figure 2:**
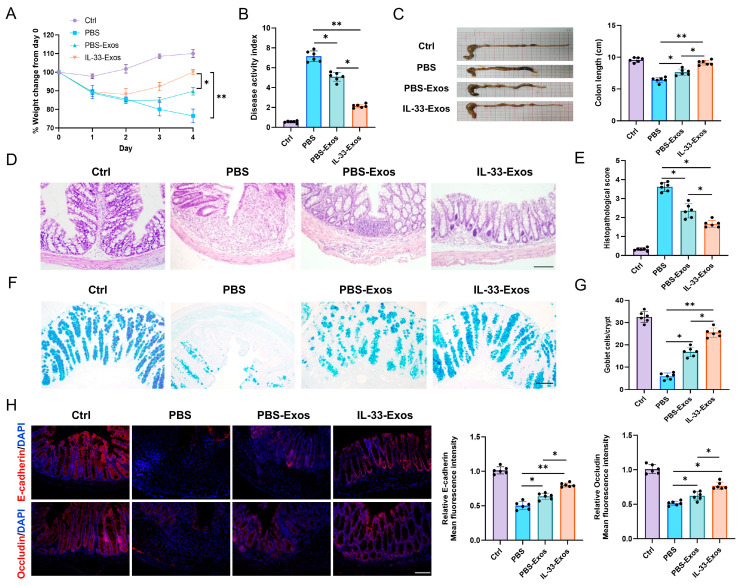
Protective effects of exosomes generated by IL-33-stimulated macrophages in TNBS-treated mice. (**A**) Changes in body weight of experimental mice in each group on days 0–4. (**B**) DAI scores on day 4. (**C**) Colon appearance and colon length on day 4. (**D**) H&E staining to assess colon tissue pathology in mice on day 4. Scale bar, 100 μm. (**E**) Statistical analysis of colonic histopathology scores. (**F**,**G**) AB-PAS staining and quantitative analysis of goblet cells. Scale bar, 100 μm. (**H**) Immunofluorescence detection of E-cadherin and Occludin expression and statistical analysis. Scale bar, 100 μm. Data are mean ± SEM (*n* = 6/group). * *p* < 0.05; ** *p* < 0.01.

To evaluate the potential protective effects of IL-33-Exos in experimental intestinal inflammation in vivo, TNBS-induced colitis was established in BALB/c mice. As anticipated, TNBS-administered mice developed severe colitis characterized by bloody diarrhea, progressive weight loss, elevated disease activity index (DAI) scores, and shortened colon length. In contrast, IL-33-Exos-treated mice exhibited rapid body weight recovery, reduced DAI scores, and increased colon length compared with both PBS and PBS-Exos control groups (Figure 2A–C). Histological examination revealed that colons from TNBS-challenged mice receiving PBS displayed transmural inflammation throughout the colonic mucosa, with ulceration, extensive inflammatory cell infiltration, goblet cell depletion, and crypt architectural disruption (Figure 2D–G). Microscopically, colons from mice treated with either IL-33-Exos or PBS-Exos showed marked attenuation of tissue damage, mucosal ulceration, and mononuclear cell infiltration, accompanied by increased goblet cell numbers. Notably, IL-33-Exos treatment provided superior histological improvement relative to PBS-Exos treatment on day 4 post-induction (Figure 2D–G). Immunofluorescence analysis further demonstrated that IL-33-Exos-treated mice displayed significantly enhanced expression of the intercellular adhesion protein E-cadherin and the tight junction protein Occludin compared with PBS and PBS-Exos groups (Figure 2H). Collectively, these findings indicate that IL-33-Exos administration effectively mitigates TNBS-induced colonic inflammatory injury in mice.

### 3.3. IL-33-Exos Facilitates the Replenishment of Damaged Intestinal Epithelial Cells

**Figure 3:**
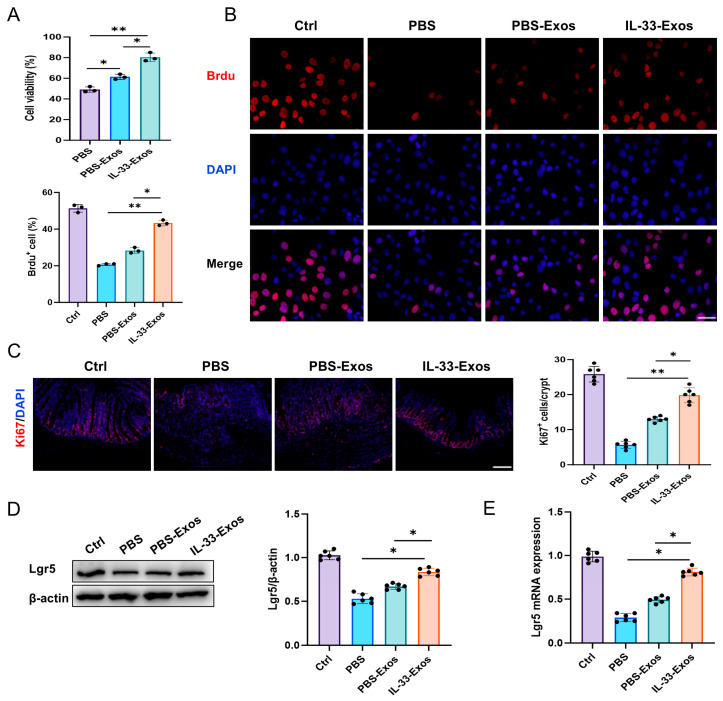
IL-33-Exos facilitate the replenishment of damaged intestinal epithelial cells. (**A**) Assessment of intestinal epithelial cell viability after TNF-α stimulation (*n* = 3/group). (**B**) Immunofluorescence detection and statistical analysis of BrdU in damaged Caco-2 cells (*n* = 3/group). Scale bar, 50 μm. (**C**) Immunofluorescence detection of Ki67 expression in intestinal epithelial cells of IBD mouse models and statistical analysis (*n* = 6/group). Scale bar, 100 μm. (**D**) Western blot detection of Lgr5 protein expression in colon tissues and statistical analysis (*n* = 6/group). (**E**) qPCR detection of Lgr5 gene expression in colon tissues (*n* = 6/group). Data are mean ± SEM. * *p* < 0.05; ** *p* < 0.01.

We next explored the effect of IL-33-Exos on the viability and proliferation of intestinal epithelial cells following inflammatory insults. The CCK-8 assay was employed to evaluate the viability of TNF-α-stimulated intestinal epithelial cells. The results demonstrated that treatment with IL-33-Exos markedly increased the viability of damaged Caco-2 cells (up to 80%), an effect that was superior to that observed with PBS-Exos at the same concentration (Figure 3A). To assess the proliferative activity of intestinal epithelial cells, a BrdU incorporation assay was performed. Immunofluorescence analysis revealed that Caco-2 cell proliferation was suppressed in the PBS group, whereas IL-33-Exos treatment significantly increased the percentage of BrdU^+^ cells compared to PBS-Exos treatment (Figure 3B). In addition, IL-33-Exos treatment obviously increased the number of Ki67^+^ intestinal epithelial cells in colon tissues of TNBS-challenged mice compared to PBS-Exos-treated colitic mice (Figure 3C). Western blot and quantitative RT-PCR analyses confirmed that IL-33-Exos markedly upregulated the expression of Lgr5, a marker of intestinal stem cells, in colon tissues of colitic mice (Figure 3D,E). Collectively, these data indicate that IL-33-Exos enhances intestinal epithelial cell proliferation and promotes the replenishment of damaged epithelial populations.

### 3.4. IL-33-Exos Improves the Integrity of the Intestinal Epithelial Barrier In Vitro

**Figure 4:**
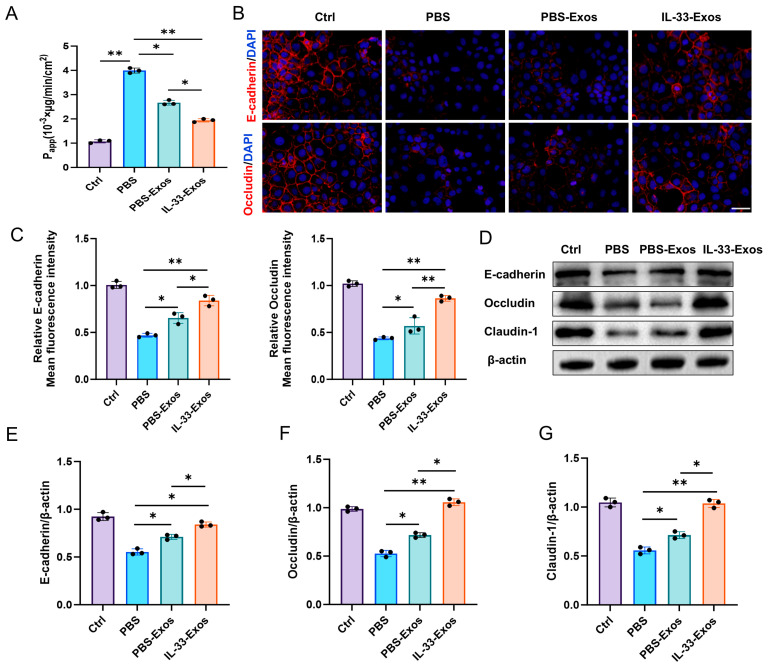
IL-33-Exos improves the integrity of intestinal epithelial cell barrier. (**A**) Assessment of intestinal epithelial cell permeability after TNF-α stimulation. (**B**,**C**) Immunofluorescence detection of E-cadherin and Occludin expression in intestinal epithelial cells and statistical analysis. Scale bar, 50 μm. (**D**–**G**) Western blot detection and statistical analysis of E-cadherin, Occludin, and Claudin-1 protein levels in intestinal epithelial cells. Data are mean ± SEM (*n* = 3/group). * *p* < 0.05; ** *p* < 0.01.

To determine how IL-33-Exos influence the intestinal epithelial barrier during inflammation, we assessed intercellular permeability and the expression of junctional proteins critical for barrier integrity. Treatment with IL-33-Exos significantly attenuated the increased permeability of Caco-2 monolayers induced by TNF-α (Figure 4A). Immunofluorescence analysis revealed that TNF-α treatment markedly reduced the expression of the intercellular adhesion protein E-cadherin and the tight junction protein Occludin in Caco-2 cells, accompanied by fragmented and discontinuous filamentous staining patterns. In contrast, IL-33-Exos significantly upregulated the expression of E-cadherin and Occludin compared to PBS-Exos, restoring continuous filamentous connections between adjacent cells (Figure 4B,C). Consistent with these findings, Western blot analysis confirmed that TNF-α stimulation decreased the expression of E-cadherin, Occludin, and Claudin-1 proteins, all of which were upregulated by IL-33-Exos treatment (Figure 4D–G). Collectively, these data demonstrate that IL-33-Exos plays an essential role in preserving the integrity of the intestinal mucosal barrier under inflammatory conditions.

### 3.5. IL-33-Exos Promotes β-Catenin Nucleus Translocation in Intestinal Epithelial Cells

**Figure 5:**
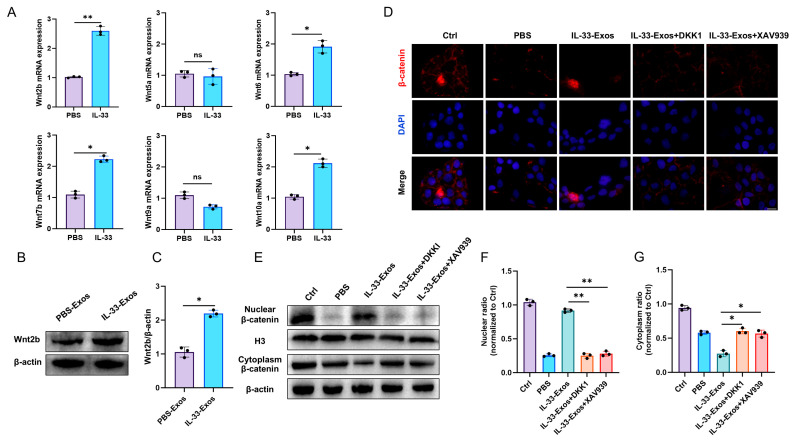
IL-33-Exos promotes β-catenin nucleus translocation in intestinal epithelial cells. (**A**) qPCR detection of the expression of Wnt ligand genes in macrophages in response to IL-33 treatment. (**B**,**C**) Western blot validation of Wnt2b protein expression and quantitative analysis. (**D**) Immunofluorescence analysis of β-catenin distribution inside and outside the cell nucleus. Scale bar, 20 μm. (**E**–**G**) Western blot detection and statistical analysis of β-catenin expression in the cytoplasm and nucleus. Data are mean ± SEM (*n* = 3/group). * *p* < 0.05; ** *p* < 0.01. ns indicates no statistical difference.

Macrophages have been shown to serve as both recipients and sources of Wnt signaling [24]. We therefore investigated whether IL-33 treatment modulates Wnt ligand gene expression in macrophages. Quantitative RT-PCR analysis revealed that IL-33 stimulation significantly upregulated the expression of intracellular Wnt2b, Wnt6, Wnt7b, and Wnt10a, but not Wnt5a and Wnt9a mRNA, in macrophages compared with the control group (Figure 5A). Furthermore, Western blot analysis confirmed the presence of Wnt2b protein in exosome samples, which was elevated following IL-33 treatment (Figure 5B,C). These findings collectively indicate that Wnt ligands produced by macrophages can be packaged into and released via exosomes.

Activation of canonical Wnt signaling triggers dissociation of β-catenin from the destruction complex, enabling its accumulation and subsequent nuclear translocation to initiate transcription of downstream target genes. DKK1 and XAV939 antagonize this pathway through distinct mechanisms. Here, we observed that TNF-α stimulation reduced nuclear β-catenin levels in epithelial cells. Conversely, IL-33-Exos treatment significantly enhanced nuclear β-catenin accumulation, whereas both DKK1 and XAV939 counteracted this effect by suppressing nuclear β-catenin protein levels (Figure 5D–G). Collectively, these findings demonstrate that IL-33 modulates Wnt ligand production in macrophages and activates the Wnt/β-catenin signaling cascade in intestinal epithelial cells via exosome-mediated delivery.

### 3.6. IL-33-Exos Modulates the Integrity of Intestinal Epithelial Cell Barrier Through Wnt/β-Catenin Pathway

**Figure 6:**
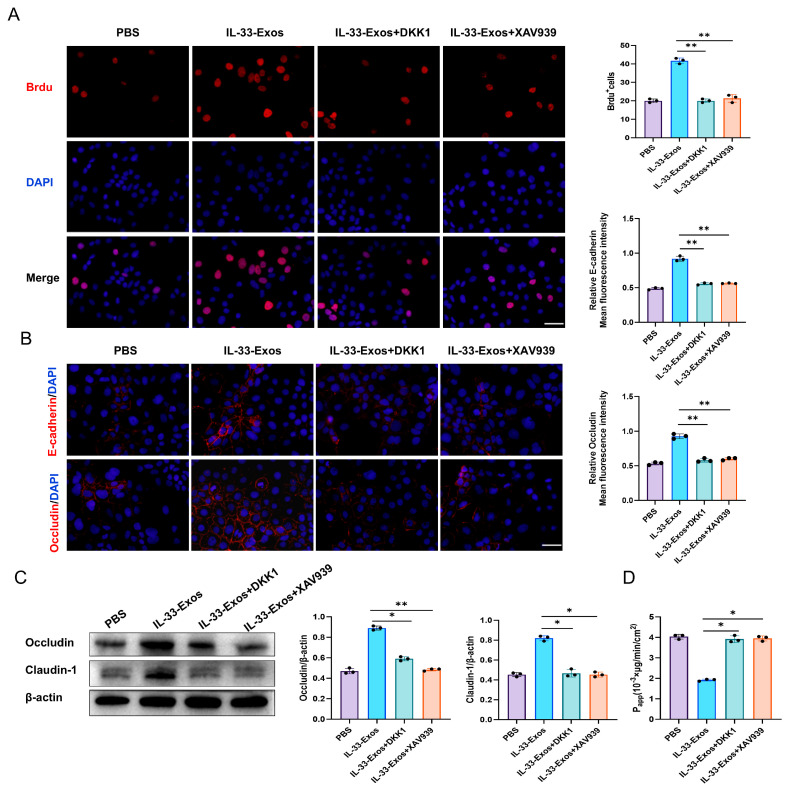
IL-33-Exos modulates the integrity of intestinal epithelial cell barrier via Wnt/β-catenin pathway. (**A**) Immunofluorescence staining of BrdU in Caco-2 cells treated with IL-33-Exos in the presence or absence of Wnt/β-catenin inhibitors, with subsequent statistical analysis. Scale bar, 50 μm. (**B**) Immunofluorescence staining and statistical analysis of E-cadherin and Occludin. Scale bar, 50 μm. (**C**) Western blot validation of Occludin and Claudin-1 expression, followed by statistical analysis. (**D**) Evaluation of intestinal epithelial cell permeability by FITC-dextran. Data are mean ± SEM (*n* = 3/group). * *p* < 0.05; ** *p* < 0.01.

Upon nuclear entry, β-catenin binds to the transcription factor TCF/LEF, thereby regulating the transcription of downstream genes associated with cell proliferation and differentiation. We subsequently investigated whether IL-33-Exos improves intestinal epithelial barrier integrity in a manner dependent on Wnt/β-catenin signaling. Compared with PBS-treated controls, IL-33-Exos administration markedly enhanced the proliferation of TNF-α-stimulated Caco-2 cells, as evidenced by a significant increase in BrdU^+^ cell numbers (Figure 6A). Immunofluorescence analysis revealed that IL-33-Exos restored the expression of impaired intercellular junction proteins, specifically E-cadherin and Occludin (Figure 6B). Western blot analysis demonstrated similar upregulation of Occludin and Claudin-1 protein levels (Figure 6C). Additionally, IL-33-Exos significantly attenuated the elevated permeability of Caco-2 cell monolayers induced by TNF-α stimulation (Figure 6D). However, blockade of Wnt/β-catenin signaling using DKK1 or XAV939 markedly inhibited the protective effects of IL-33-Exos on barrier function in damaged Caco-2 cells (Figure 6). Collectively, these data demonstrate that IL-33-Exos facilitate the restoration of barrier function in damaged intestinal epithelial cells through activation of Wnt/β-catenin signaling.

### 3.7. Blockade of Wnt/β-Catenin Signaling Inhibits the Beneficial Effect of IL-33-Exos in TNBS-Induced Colitis

**Figure 7:**
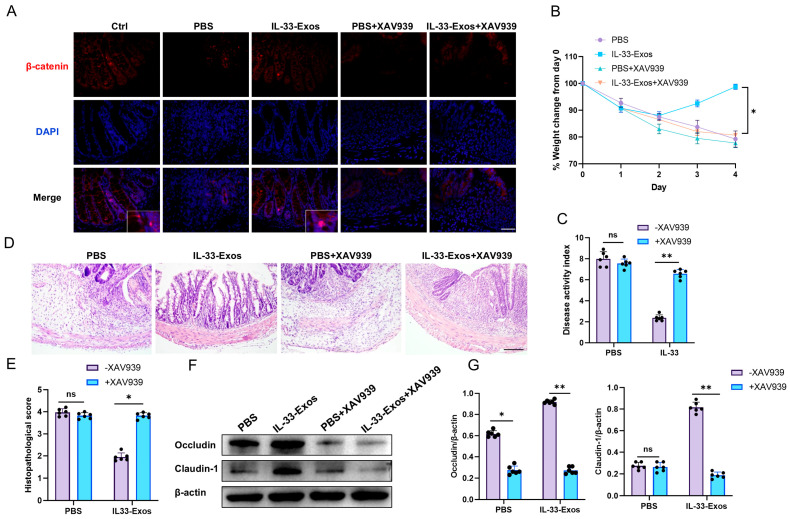
Blockade of β-catenin signaling inhibits the beneficial effect of IL-33-Exos in TNBS-induced colitis. (**A**) Immunofluorescence detection of β-catenin distribution in the cell nucleus. Scale bar, 100 μm. (**B**) Changes in body weight of mice on days 0–4 following treatment with the XAV939 inhibitor. (**C**) DAI score. (**D**) H&E staining. Scale bar, 100 μm. (**E**) Statistical analysis of colonic histopathology scores. (**F**,**G**) Western blot validation of Occludin and Claudin-1 expression and statistical analysis. Data are mean ± SEM (*n* = 6/group). * *p* < 0.05; ** *p* < 0.01. ns indicates no statistical difference.

To determine whether IL-33-Exos-mediated protection in colitis is dependent on Wnt/β-catenin signaling, XAV939 was administered in a murine TNBS colitis model. Immunofluorescence analysis revealed that IL-33-Exos promoted nuclear translocation of β-catenin from the cytoplasm in intestinal epithelial cells. This nuclear accumulation was markedly blocked by XAV939 treatment (Figure 7A). Concurrently, the therapeutic benefits of IL-33-Exos including prevention of weight loss, restoration of intestinal mucosal integrity, and attenuation of inflammatory cell infiltration were effectively abolished by XAV939 co-administration (Figure 7B–E). In line with these findings, Western blot analysis confirmed that XAV939 suppressed the IL-33-Exos-induced upregulation of tight junction proteins Occludin and Claudin-1 (Figure 7F,G). Collectively, these results demonstrate that IL-33-Exos attenuates TNBS-induced experimental colitis through activation of the Wnt/β-catenin signaling pathway.

## 4. Discussion

In the present study, we demonstrated that exosomes derived from IL-33-preconditioned macrophages markedly ameliorated inflammatory intestinal mucosal injury and promoted the restoration of intestinal barrier function. Furthermore, the beneficial effects of IL-33-Exos were implicated in upregulation of Wnt/β-catenin signaling in intestinal epithelial cells. These findings suggest that IL-33-induced macrophages function as a conduit for therapeutic signals, transmitting them to the intestinal epithelium through an exosome-dependent mechanism.

Despite their minute size, exosomes are characterized by their potent biological activity and their important role as mediators of intercellular signaling. The protective effects of exosome-based therapies for colitis have been demonstrated following intraperitoneal administration, with subsequent uptake by intestinal epithelial cells, providing a basis for the development of novel exosome-based therapeutic strategies. Previous studies have reported that exosomes derived from mesenchymal stromal cells can be internalized by CT26 mouse colon epithelial cells, resulting in augmentation of S-phase and G2-phase cell cycle progression and promotion of epithelial regeneration [25]. Additionally, exosomes derived from human umbilical cord mesenchymal stem cells (HucMSC-exo) have been shown to activate the Wnt/β-catenin signaling pathway through the transfer of key microRNAs, thereby accelerating intestinal stem cell (ISC) regeneration and intestinal epithelial repair to promote mucosal healing in colitis [26]. To date, the majority of in vitro studies investigating the effects of various agents on intestinal epithelium have employed simple monolayer cultures, such as the Caco-2 cell line, which has been extensively validated as a model of intestinal epithelial cells [27]. In this study, in vivo administration of IL-33-Exos attenuated intestinal inflammatory cell infiltration and crypt destruction, resulting in enhanced intestinal epithelial integrity. These findings indicate that IL-33-Exos plays an important role in orchestrating epithelial cell proliferation and suppression of intestinal epithelial damage in murine colitis.

Intestinal mucosal healing should be regarded as a comprehensive histological process characterized by the resolution of inflammation, epithelial proliferation and differentiation, and ultimately restoration of mucosal function [28]. In patients with IBD, intestinal epithelial damage and inflammatory responses are critically regulated by TNF-α. Therapeutic neutralization of TNF-α has been demonstrated to reduce pathogen invasion, attenuate cellular apoptosis, and preserve mucosal barrier integrity [29]. The maintenance of intestinal epithelial barrier integrity is fundamentally dependent upon robust intestinal epithelial cell renewal, wherein Lgr5^+^ ISCs differentiate into the mature cell types required for normal gastrointestinal function. Consequently, the regenerative capacity of ISCs is imperative for effective healing of intestinal epithelial mucosa in IBD. Macrophages have been demonstrated to promote the regeneration of intestinal epithelial and immune cells in murine models of colitis [12,30,31]. In this study, we found that IL-33-Exos significantly enhanced Caco-2 cell viability and proliferation, as evidenced by increased BrdU-positive cells under inflammatory conditions. The upregulation of Lgr5 protein and mRNA levels in colon tissues indicates that IL-33-Exos increase the expression of an intestinal stem cell-associated marker. Furthermore, IL-33-Exos treatment reversed TNF-α- and colitis-induced downregulation of junctional proteins, reduced epithelial permeability, and suppressed the decrease in mucus-associated goblet cell numbers. These findings reveal that IL-33-induced macrophages release exosomes to enhance intercellular communication with intestinal epithelial cells, thereby suppressing epithelial damage and maintaining barrier function.

Wnt signaling plays a crucial role in determining cell fate and establishing tissue patterning during normal development, as well as facilitating tissue repair following injury. Macrophages express Wnt signaling receptors and serve as important sources of Wnt ligands [24]. Owing to their remarkable plasticity, macrophages exhibit phenotypes associated with selective expression of specific Wnt ligands. Wnt1, Wnt2b, Wnt3a, Wnt7b, and Wnt10a are predominantly released by M2 macrophages [32,33]. STAT6 mediates the expression of canonical Wnt ligands in M2a macrophages, thereby activating Wnt signaling in intestinal epithelial cells. The specific Wnt ligand identity determines whether signaling occurs through β-catenin-dependent or -independent pathways. The majority of Wnt ligands activate β-catenin signaling, whereas a minor subset antagonizes β-catenin signaling and/or functions independently of it [34,35]. Consistent with these findings, we observed that IL-33 upregulated mRNA expression of Wnt2b, Wnt6, Wnt7b, and Wnt10a in macrophages. Among these Wnt ligands, Wnt2b and Wnt10a are associated with canonical Wnt signaling.

Recent studies have revealed that Wnt proteins can be secreted into the microenvironment via extracellular vesicles or exosomes. In the intestines of healthy mice, Wnt2b is widely distributed throughout the mucosal layer, lamina propria, and intestinal epithelial cells, whereas Wnt10a expression is restricted to the mucosal layer and lamina propria [33], suggesting that Wnt2b may play a particularly important role in intestinal epithelial cells. In this study, Western blot analysis demonstrated that the relative abundance of Wnt2b in IL-33-Exos was approximately twofold higher than that in PBS-Exos. Wnt2b, derived from intestinal epithelial cells, plays a critical role in intestinal regeneration by inducing proliferation of the Tert^+^ quiescent intestinal stem cell subpopulation [36]. Wnt2b, a secreted protein produced by stromal and mesenchymal cells, has been shown to promote ISC regeneration and functional repair of intestinal epithelial cells following injury [37,38]. Comparative analysis of Wnt ligand expression patterns released by M2-type macrophages revealed that the basal expression level of Wnt2b in normal intestine exceeds that of other ligands.

When exosome-associated Wnt proteins interact with target cells, they can activate the Wnt signaling pathway, thereby regulating cellular biological functions and behaviors [39]. Exosomes derived from intestinal fibroblasts have been shown to play a critical role in establishing the ISC niche by transmitting Wnt and epidermal growth factor (EGF) activity [40]. To test the hypothesis that canonical Wnt ligands, such as Wnt2b, carried by IL-33-Exos activate β-catenin signaling, we employed Wnt receptor inhibitors in this study. DKK1 functions as a secreted antagonist of Wnt proteins by competitively binding to the LRP5/6 co-receptor, thereby impeding downstream target gene expression [41,42]. Our data showed that DKK1 treatment abrogated IL-33-Exos-mediated β-catenin nuclear translocation in Caco-2 cells. Furthermore, DKK1 suppressed intestinal epithelial cell proliferation and increased cell permeability, indicating that Wnt proteins released by IL-33-stimulated macrophages play an important role in ameliorating inflammatory injury. The ultimate outcome of Wnt signaling is determined by genes whose transcription is regulated by β-catenin and TCF [43]. Pharmacological inhibition of β-catenin signaling by XAV939 attenuated the protective effects of IL-33-Exos in experimental colitis, as evidenced by impaired nuclear β-catenin accumulation, disrupted junctional integrity, and aggravated intestinal mucosal damage.

## 5. Conclusions

The present study reveals a potential role of IL-33-Exos in maintaining intestinal mucosal homeostasis in TNBS-induced colitis. IL-33-induced macrophages serve as a source of Wnt ligands, which are transferred via exosomes to contribute to Wnt/β-catenin signaling in epithelial cells, thereby mitigating intestinal mucosal injury and improving barrier function. These findings suggest that IL-33-Exos may promote epithelial repair in experimental colitis, offering a novel therapeutic avenue for clinical management of inflammatory bowel disease.

## Data Availability

The original contributions presented in this study are included in the article. Further inquiries can be directed to the corresponding authors.
